# Presence of multiple lesion types with vastly different microenvironments in C3HeB/FeJ mice following aerosol infection with *Mycobacterium tuberculosis*

**DOI:** 10.1242/dmm.019570

**Published:** 2015-06-01

**Authors:** Scott M. Irwin, Emily Driver, Edward Lyon, Christopher Schrupp, Gavin Ryan, Mercedes Gonzalez-Juarrero, Randall J. Basaraba, Eric L. Nuermberger, Anne J. Lenaerts

**Affiliations:** ^1^ Mycobacteria Research Laboratories, Department of Microbiology, Immunology and Pathology, Colorado State University, Fort Collins, CO 80523, USA; ^2^ Center for Tuberculosis Research, Department of Medicine, Johns Hopkins University School of Medicine, Baltimore, MD 21231, USA

**Keywords:** C3HeB/FeJ, Tuberculosis, Mouse models, Chemotherapy, Neutrophil

## Abstract

Cost-effective animal models that accurately reflect the pathological progression of pulmonary tuberculosis are needed to screen and evaluate novel tuberculosis drugs and drug regimens. Pulmonary disease in humans is characterized by a number of heterogeneous lesion types that reflect differences in cellular composition and organization, extent of encapsulation, and degree of caseous necrosis. C3HeB/FeJ mice have been increasingly used to model tuberculosis infection because they produce hypoxic, well-defined granulomas exhibiting caseous necrosis following aerosol infection with *Mycobacterium tuberculosis*. A comprehensive histopathological analysis revealed that C3HeB/FeJ mice develop three morphologically distinct lesion types in the lung that differ with respect to cellular composition, degree of immunopathology and control of bacterial replication. Mice displaying predominantly the fulminant necrotizing alveolitis lesion type had significantly higher pulmonary bacterial loads and displayed rapid and severe immunopathology characterized by increased mortality, highlighting the pathological role of an uncontrolled granulocytic response in the lung. Using a highly sensitive novel fluorescent acid-fast stain, we were able to visualize the spatial distribution and location of bacteria within each lesion type. Animal models that better reflect the heterogeneity of lesion types found in humans will permit more realistic modeling of drug penetration into solid caseous necrotic lesions and drug efficacy testing against metabolically distinct bacterial subpopulations. A more thorough understanding of the pathological progression of disease in C3HeB/FeJ mice could facilitate modulation of the immune response to produce the desired pathology, increasing the utility of this animal model.

## Abstract (precis)

**Summary:** C3HeB/FeJ mice develop three morphologically distinct lesion types, which differ with respect to bacterial load, cellular composition and degree of immunopathology following low-dose aerosol infection with *Mycobacterium tuberculosis*.

## INTRODUCTION

It has been estimated that approximately one third of the world is infected with *Mycobacterium tuberculosis*, with the vast majority of these individuals serving as a latently infected asymptomatic reservoir (Lin and Flynn, 2010). Since 2008, the incidence of multiple-drug-resistant (MDR) tuberculosis (TB) in Africa has almost doubled, while in southeast Asia the incidence has increased more than 11 times (World Health Organization, 2013). Globally, the incidence of MDR TB increased 42% from 2011 to 2012, with almost 10% of those cases being extensively drug-resistant (XDR) TB. With the rise in MDR and XDR TB, new drugs and especially drugs with a novel mechanism of action or drugs that can shorten the duration of treatment are desperately needed.

In humans, TB presents as a diverse spectrum of disease (Laennec, 1823). Historically, it took the discovery of the tubercle bacillus and development of diagnostic staining methodologies to definitively ascertain that the diverse manifestations of this disease were the result of a single infectious agent. Histopathological studies revealed that, even within the lungs of a single individual, multiple lesion types coexist (Canetti, 1955). Through the use of ^18^F-fluorodeoxyglucose positron emission tomography (PET) computed tomography (CT), it has recently become apparent that individual pulmonary lesions can change substantially over time (Barry et al., 2009). Importantly, these observations have been confirmed and extended using the nonhuman primate model, suggesting that lesions within an individual animal follow independent pathological trajectories and that the sum of these trajectories might best represent disease outcome (Lin et al., 2013, 2014). Although nonhuman primates can replicate many of the lesion types and histological features of human disease, the high cost precludes their use for drug screening.

The ultimate goal of TB drug development programs is to shorten the duration of therapy needed to cure disease, prevent the emergence of drug resistance and phenotypic drug tolerance, and target difficult-to-treat persistent subpopulations of bacilli. Small animal models of TB infection are an important component of drug screening efforts and represent a cost-effective means of accelerating preclinical drug development. In order to obtain results that have predictive utility for human clinical trials, small animal models should replicate the pathophysiological conditions believed to exist within human pulmonary TB lesions. Important parameters include: (1) the production of caseous necrosis accompanied by collagen encapsulation, which can influence drug penetration and the nutrient supply available to bacilli (Dartois, 2014); (2) hypoxia, which can influence the metabolic state of *M. tuberculosis* bacilli (Wayne and Hayes, 1996); and (3) intracellular as well as extracellular populations of bacilli, which can impact drug efficacy (Grosset, 2003). Although BALB/c and C57BL/6 mouse strains have been extensively used for modeling aerosol infection with TB, these strains present with a single morphological lesion type following low-dose aerosol (LDA) infection with virulent *M. tuberculosis* (Rhoades et al., 1997), and therefore do not represent the full range of disease manifestations observed in affected humans. The cellular, inflammatory granulomas in these strains of mice fail to become hypoxic (Driver et al., 2012; Harper et al., 2012) and might not produce the microenvironmental conditions needed to generate metabolically distinct subpopulations of bacteria, especially persistent organisms that are difficult to treat. In addition, the majority of the bacilli are located intracellularly (Hoff et al., 2011), which could potentially bias drug screens by overestimating the efficacy of compounds that accumulate within macrophages. It has become apparent that the caseum of mature granulomas might substantially impede drug penetration, limiting *in vivo* drug efficacy (Prideaux et al., 2011). Mouse strains that do not exhibit this aspect of the granulomatous process might fail to accurately model the effects of drug penetration and activity under specific microenvironments, aspects that are crucial components of *in vivo* drug efficacy (Lanoix et al., 2015; Dartois, 2014).

**TRANSLATIONAL IMPACT**

**Clinical issue**

Tuberculosis (TB) is a pulmonary (lung) disease spread by aerosol transmission. Around 2-billion people worldwide are infected with *Mycobacterium tuberculosis*, the bacterium that causes TB. Once infected, approximately 10% of individuals rapidly develop active TB, which is characterized by fever, weight loss and productive cough. However, 90% of infected individuals contain and control the infection. Although these individuals are asymptomatic and noncontagious, they represent a reservoir of potential disease and a proportion of them subsequently present with active disease. Reactivation of TB is typically associated with an age-related decline in immunity, immunosuppression or immune-compromising diseases such as HIV. With the emergence of multi-drug-resistant TB, new drugs and chemotherapeutic regimens are desperately needed to treat the disease. Although mouse models have proven useful for TB drug discovery, many mouse strains fail to accurately reproduce the pulmonary pathology observed in human disease.

**Results**

Here, the authors investigate the histopathological response to aerosol infection in the C3HeB/FeJ mouse model, which develops caseous necrotic pulmonary granulomas, a type of lung lesion often seen in humans with TB. A comprehensive pathological evaluation reveals that three morphologically distinct lesion types emerge following infection, in contrast to the more commonly used mouse strains, which only present with a single lesion type. Notably, the bacterial load varies between lesion types and this variability reflects differences in the immunological control of bacterial replication. Finally, the authors identify a single lesion type that is responsible for the significant early mortality observed in a subpopulation of C3HeB/FeJ mice following infection.

**Implications and future directions**

Cost-effective small animal models that represent the full spectrum of human TB are needed to augment drug development efforts, evaluate novel multi-drug combination regimens and prevent the emergence of drug resistance. Although the C3HeB/FeJ mouse model has shown promise, variability in pulmonary colony-forming unit (CFU) counts and early mortality have complicated its usage. The realization that three distinct lesion types with different bacterial loads can emerge following aerosol infection of C3HeB/FeJ mice will allow researchers to understand and properly interpret the variability of pulmonary bacterial counts typically observed when using C3HeB/FeJ mice as a model for TB. Moreover, a more complete understanding of the pathological process that underlies lesion development in the lungs will increase the reproducibility, and therefore the utility, of this animal model. Immunomodulatory and chemotherapeutic strategies are presently being investigated to reduce early mortalities while preserving the caseous necrotic lesions that make this animal model relevant to human TB.

Mouse models that display more realistic pulmonary pathology might be more predictive of drug activity in human clinical trials. C3HeB/FeJ mice (also known as the Kramnik mouse model) generate hypoxic, caseous necrotic pulmonary lesions with abundant intracellular and extracellular bacteria following LDA infection with *M. tuberculosis*, and therefore represent an attractive model in which to study TB drug activity. In support of this, previous work from our laboratory demonstrated that clofazimine (CFZ) had significant activity in BALB/c mice and that this activity was highly attenuated in C3HeB/FeJ mice (Irwin et al., 2014). The attenuation observed for CFZ was shown to be dependent upon the pathological progression of disease, because CFZ had significant activity in C3HeB/FeJ mice when treatment was initiated prior to the formation of well-defined pulmonary granulomas. The differential activity of CFZ in a mouse model that reproduces the complex pathological manifestation of granulomatous disease underscores the central role of the pathological process in assessing *in vivo* drug efficacy.

Owing to the crucial role of the pathological response in the lungs in TB, a more comprehensive understanding of the histopathological response to aerosol infection in C3HeB/FeJ mice was warranted. In these experiments, we characterized the pathological progression of lesion development over time following an LDA infection and examined the cellular composition and distribution of cell types in the resulting granulomas. We identified three distinct lesion types that arose following LDA infection. Importantly, the heterogeneity of lesion types represented differing levels of host control of bacterial replication and of the progressive inflammatory response as evidenced by the number of bacteria and morphometric analysis of lesion size over time. The exceptionally large number of bacilli characteristic of C3HeB/FeJ mice coupled with the highly sensitive SYBR Gold staining methodology allowed us to easily visualize the location of the bacilli, which is normally quite challenging in most animal models (Hoff et al., 2011; Manabe et al., 2008; Rayner et al., 2013; Turner et al., 2003) and in humans (Canetti, 1955) owing to the low number of bacilli present in the lungs. Finally, we correlated rapid pulmonary neutrophilia with progressive consolidation of lung parenchyma that led to the early mortality observed in a proportion of C3HeB/FeJ mice, underscoring the damaging role of an excessive neutrophilic response in the lungs.

## RESULTS

### Bacterial replication and C3HeB/FeJ morbidity/mortality following aerosol infection

**Fig. 1.**
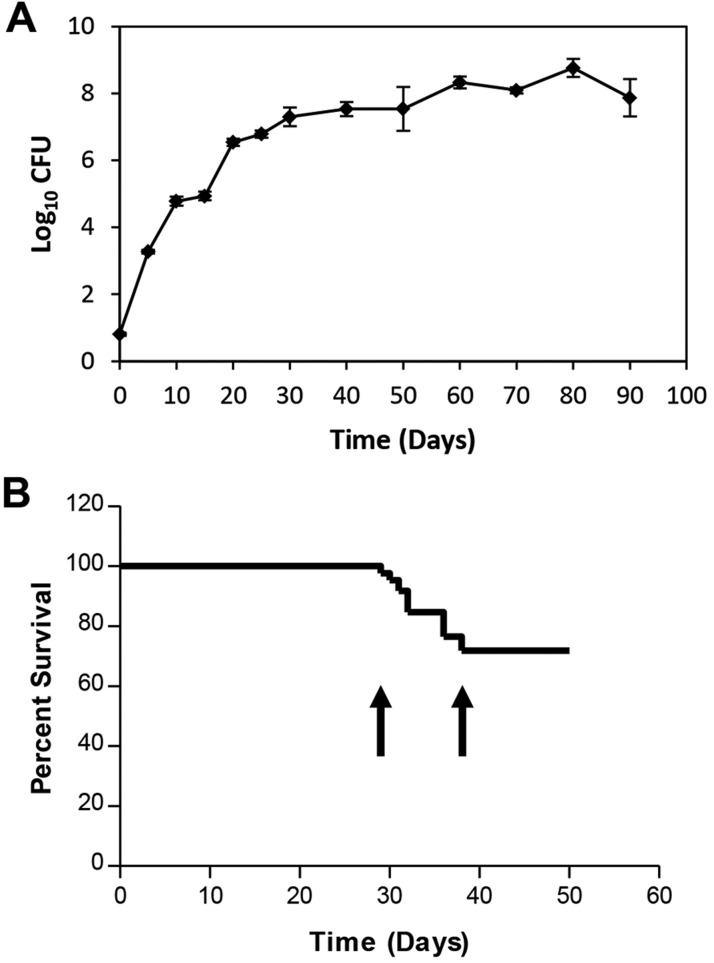
**Kinetics of bacterial replication and effects on morbidity and mortality of C3HeB/FeJ mice.** (A) Bacterial growth curve in the lungs of C3HeB/FeJ mice exposed to an LDA of *M. tuberculosis* Erdman. Data points represent mean log_10_ CFU±s.e.m. of five animals per time point from one representative experiment. (B) A separate experiment showing the survival curve and characteristic window of mortality (arrows) of C3HeB/FeJ mice (*n*=85) following aerosol infection.

Following LDA infection of C3HeB/FeJ mice using 50-75 colony-forming units (CFU) of *M. tuberculosis* Erdman, bacterial numbers within the lung rapidly increased to more than 7 log_10_ CFU by 30 days post-infection (Fig. 1A). The increase in bacterial numbers in the lung slowed after 30 days, culminating in an additional 0.6 log_10_ CFU increase between 30 and 90 days.

In a series of experiments, we measured mouse survival over time. A consistent pattern emerged whereby significant mortality was observed between 28-45 days after LDA; however, mice that survived beyond 45 days generally survived at least 14 weeks with only minimal mortality observed in the intervening time (Fig. 1B). Of importance, although the timing of this window of mortality remained fairly constant across multiple experiments, the percentage of mortality within this window varied significantly between multiple experiments, ranging from 10 to 40% (data not shown).

### C3HeB/FeJ mice developed three distinct pulmonary lesion types following aerosol infection

We next performed a comprehensive pathological examination of the pulmonary lesions that arose following LDA infection in the C3HeB/FeJ mice. Interestingly, upon histological examination we were able to identify three distinct lesion types by 5 weeks following aerosol infection that differed by cellular composition and organization. For descriptive efficiency, we arbitrarily identified these lesions as Type I, Type II or Type III.

**Fig. 2.**
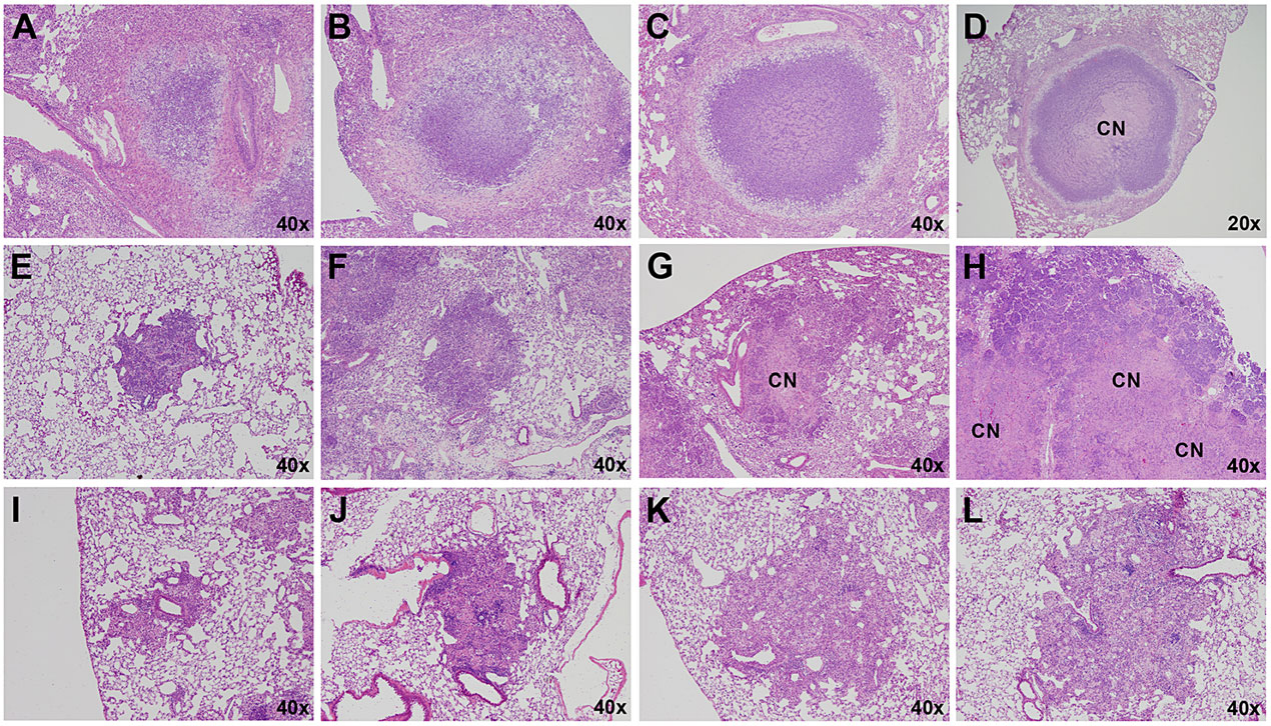
**Progression of Type I, II and III lesions over time.** Each panel represents a single lesion (H&E-stained) obtained from an individual representative animal (*n*=5) euthanized at the time point defined here. Progression of Type I lesions from individual animals at 45 (A), 50 (B), 55 (C) and 61 (D) days following aerosol infection. Progression of Type II lesions from individual animals at 20 (E), 35 (F), 38 (G) and 40 (H) days following aerosol infection. Progression of Type III lesions from individual animals at 30 (I), 35 (J), 61 (K) and 75 (L) days following aerosol infection. CN, caseous necrosis.

**Fig. 3.**
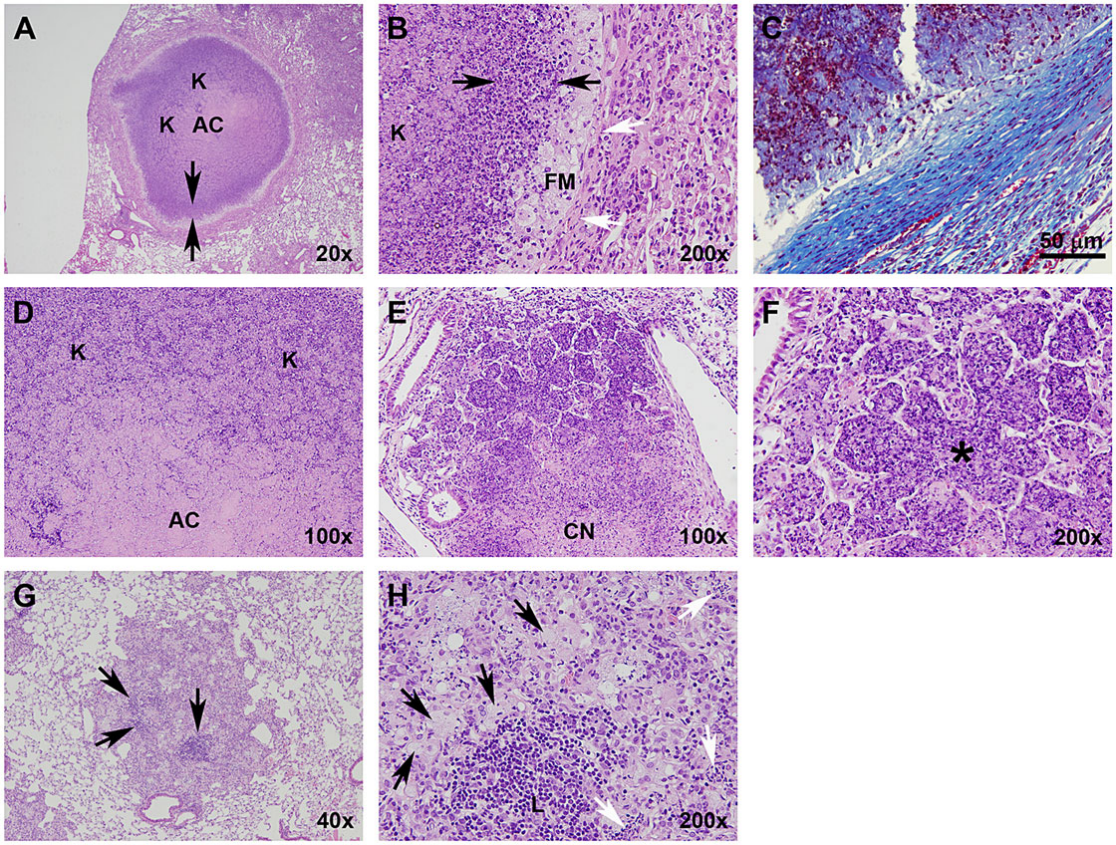
**Type I, II and III lesions vary with respect to their cellular composition, organization and collagen encapsulation.** (A) Type I granuloma showing the acellular caseum, distinct band of darkly stained intact neutrophils (arrows delineate margins), primarily neutrophil-derived zone of karyorrhectic debris, lightly stained foamy macrophage rim, and collagen encapsulation. (B) Higher-magnification image of A illustrating a zone of intact neutrophils (black arrows), foamy macrophage rim and collagen encapsulation (white arrows). (C) Masson's Trichrome stain for collagen along the peripheral margin of a Type I lesion. (D) Interior region of a Type I granuloma, showing the acellular caseum and karyorrhectic debris. (E) Type II lesion with a caseous necrotic interior, and extensive consolidation of peripheral alveoli. (F) Pulmonary acinus (*) completely occluded with neutrophils and cellular debris. (G) Type III lesion with distinct lymphocytic clusters (arrows). (H) Higher-magnification image of G showing foamy macrophages (black arrows) and a lymphocytic cluster (L). Small isolated pockets containing small numbers of neutrophils (white arrows) are occasionally present, especially at later time points as foamy macrophages undergo necrosis. AC, acellular caseum; K, karyorrhectic debris; FM, foamy macrophages; CN, central necrosis. H&E staining unless otherwise noted.

Type I lesions (Fig. 2A) most closely resembled classical human TB granulomas in that they were solid, encapsulated caseous necrotic lesions. These granulomas were initially identified by Igor Kramnik's group (Pichugin et al., 2009) and have been described previously (Driver et al., 2012). These lesions became evident 35-45 days following LDA, and originated as a focal accumulation of foamy macrophages interspersed with neutrophils, often proximal to a bronchus. The peripheral margins contained abundant, loosely aggregated epithelioid macrophages interspersed with a small number of scattered lymphocytes (Fig. 2A). As this lesion morphology progressed, the number of neutrophils increased rapidly, and the beginning of a dense central neutrophilic core was evident, variably surrounded by smaller regions of loosely packed neutrophils and foamy macrophages (Figs 2B and 3A,B). The epithelioid macrophages immediately adjacent to the neutrophilic core stained less intensely with eosin, and it appeared that these cells were transforming into foamy macrophage cells. Loosely packed epithelioid macrophages, activated macrophages and a small number of lymphocytes composed the peripheral extremity of the lesion. By 7-10 weeks following infection, the Type I granulomas took on a highly organized appearance, composed of a densely packed neutrophilic core, with or without central caseation (Fig. 2C). This core region was surrounded by a rim of diffusely stained foamy macrophages, encapsulated by a fibrotic rim (Fig. 3C). The peripheral margin of the granuloma was composed of fibroblasts, epithelioid and activated macrophages, and a small number of scattered lymphocytes. At this time, multiple coalescing Type I granulomas were occasionally observed. After 8-10 weeks, Type I granulomas continued to progressively enlarge, although the cellular composition did not change. However, the central region of the neutrophilic core progressively degenerated into an acellular homogeneous caseum diffusely stained by eosin (Fig. 2D). Initially, alveolar septae were clearly visible and retained the structural appearance of the lung. Gradually, as neutrophils within individual alveoli degenerated as evidenced by punctate karyorrhectic debris, the structure of the interalveolar septae within the central region of the granuloma began to degenerate into isolated islands as previously described (Driver et al., 2012). Ultimately, the karyorrhectic debris and even the septal wall fragments completely degenerated until no histologically identifiable lung structure was evident (Fig. 3D). This degeneration typically originated within the centermost region and progressed outward as the granuloma enlarged. The periphery of the core region still retained a distinct band of intact neutrophils within the collagen rim.

Type II lesions resembled a rapidly progressive, granulocytic pneumonia composed almost entirely of neutrophils. By 20 days following LDA, small cellular aggregates composed of activated and epithelioid macrophages and aggregates of neutrophils that had completely impacted the alveoli were evident (Fig. 2E). By day 25, the beginning stage of rapidly progressive neutrophilia was evident as fulminant necrotizing alveolitis (Fig. 2F). A distinct central region of epithelioid macrophages was surrounded by a large rim of neutrophils that appeared to be expanding outward by progressively infiltrating adjacent alveoli. Small aggregates of lymphocytes were occasionally observed, but were confined to the extreme periphery of the lesion boundary. The initial stages of cellular necrosis were evident at this time, particularly within the central region. By 30 days post-infection, the majority of Type II lesions had substantially increased in size and contained a central region of caseous necrosis (Figs 2G and 3E). The central region had evidence of cellular necrosis, karyorrhectic debris, and fragmentation and degeneration of septal walls, similar to the caseous necrotic response observed in the center of Type I lesions (although at a much earlier time point than the Type I lesions). Notably, few if any lymphocytes were observed in the vicinity of this lesion type and, in mice in which this lesion type predominated, lymphocytes were notably absent in other areas of the lung. Inflammatory exudate, primarily composed of edematous fluid, neutrophilic debris and foamy macrophages, frequently consolidated terminal bronchioles, often leading to complete occlusion. By 40 days after infection, rapidly progressive neutrophilia resulted in large areas of consolidation of lung parenchyma that radiated outwards over time (Figs 2H and 3F). The central areas of these lesions exhibited the various stages of caseous necrosis as described above, with large areas of amorphous eosin-stained material with no visible cellular structure. This lesion type was rapidly progressive, and culminated in complete consolidation of large areas of lung and collapse of the lung parenchyma along the exterior edge of the lesion. Complete consolidation of entire lung lobes was frequently observed. Although Type II lesions were similar to the Type I granulomas in that they were primarily neutrophil-dominated, they lacked the highly organized fibrotic structure and collagen deposition diagnostic of encapsulated Type I granulomas.

Type III lesions in C3HeB/FeJ mice were cellular, inflammatory lesions that were indistinguishable from pulmonary lesions typically observed in BALB/c (Hoff et al., 2011) and C57BL/6 (Rhoades et al., 1997) mice. Briefly, Type III lesions were initially composed primarily of mononuclear phagocytes and activated macrophages, located proximally to a blood vessel with mild alveolitis (Fig. 2I). By 35 days post-infection, large numbers of epithelioid macrophages were evident, admixed with activated macrophages and large numbers of lymphocytes typically arranged in perivascular and peribronchiolar cuffs that exhibited mild to moderate interstitial fibrosis and alveolar thickening (Figs 2J and 3G). Small isolated pockets of neutrophils were occasionally present (Fig. 3H), usually confined to localized regions within a lesion. Inflammatory exudate was sometimes apparent within bronchioles, but rarely resulted in complete occlusion. By 55 days, many of the epithelioid-like macrophages had transformed into foamy macrophages (Figs 2K and 3H). Large numbers of lymphocytes were found both in large aggregates and interspersed throughout the lesion, often in association with macrophages. By 75 days following infection, large numbers of foamy macrophages (Figs 2L and 3H) containing abundant lipid vesicles were present. Localized individual cellular necrosis with associated punctate karyorrhectic debris occurred primarily within foamy macrophages, and resulted in microcavities containing necrotic debris and variably small numbers of neutrophils within otherwise densely packed cellular lesions.

### C3HeB/FeJ mice with predominantly Type II lesions had decreased survival

**Fig. 4.**
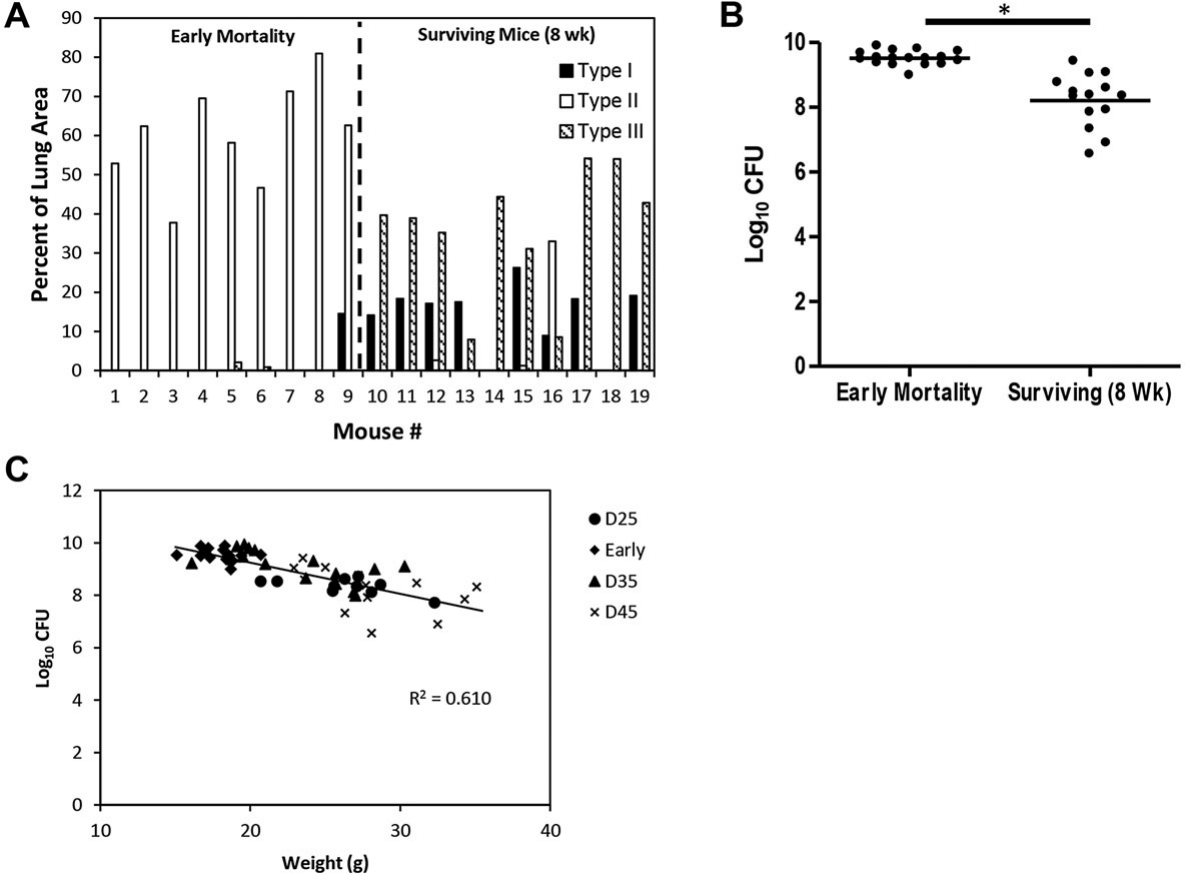
**Early-mortality mice displayed primarily Type II lesions, and had higher pulmonary bacterial loads and lower body weights.** (A) Early-mortality mice primarily exhibited Type II lesions, whereas the surviving mice exhibited primarily Type I and Type III lesions. Lesion area analysis was performed on H&E-stained histological sections of all five lung lobes from individual mice (*n*=9 for early mortality mice and *n*=10 for surviving mice). Early-mortality mice represent mice euthanized between day 30 and 41 post-infection owing to morbidity. Surviving mice were euthanized 8 weeks following infection. Data are expressed as the percentage of the total lung area (all five lobes) for each lesion type compared to the total lung area for individual mice. (B) Early-mortality mice (*n*=17) had a higher pulmonary bacterial load compared to surviving mice (*n*=14). Data represent the logarithms of serial dilutions of lung homogenates obtained from whole lungs of individual mice. Statistical comparison performed using Student's *t*-test. *P<0.0001. (C) Animal body weight was inversely correlated with pulmonary bacterial burden. Terminal body weight was taken immediately prior to euthanasia with pulmonary bacterial load expressed as log_10_ CFU. Early-mortality mice were euthanized between 30 and 41 days post-infection owing to morbidity, and ten mice were euthanized at 25 (D25), 35 (D35) and 45 (D45) days post-infection.

We next examined the mice that had to be euthanized prior to experimental endpoints to adhere to Colorado State University's Institutional Animal Care and Use Committee (IACUC) guidelines (loss of greater than 20% body weight, unthrifty appearance, etc.). This group of mice exhibited significant morbidity in multiple experiments within a highly reproducible window (28-45 days after infection). Morphometric analysis of the total lesion area occupied by each of the three lesion types in comparison to the total lung area was performed on hematoxylin and eosin (H&E)-stained whole lung sections to quantify disease severity. Analysis of the early-mortality mice (mice humanely euthanized between days 30 and 41 in this particular experiment) indicated that 100% of these animals had predominantly Type II lesions composed of fulminant neutrophilia (Fig. 4A) that occupied large areas of the lung parenchyma and resulted in observable weight loss, compromised respiratory function and a decline in overall health of the affected animals. In contrast, of the mice that survived to 8 weeks, all had a mixture of Type I and Type III lesions, with little evidence of Type II lesion involvement. Of the three surviving mice that had detectable Type II lesions, only one mouse (Fig. 4A, mouse 16) had a significant percentage of lung area occupied by Type II lesions, although it should be noted that the area occupied by Type II lesions in this mouse was approximately half that observed in the early-mortality mice.

Pulmonary bacterial load was assessed by plating serial dilutions of whole-lung homogenates on 7H11 agar. Seventeen mice displaying signs of morbidity substantial enough to warrant humane euthanasia (early-mortality mice) were selected for CFU determination. All of these mice were euthanized between 30-41 days post-infection in this experiment. Fourteen surviving mice were euthanized 8 weeks post-infection (Fig. 4B). The early-mortality mice had 1.3 log_10_ CFU more bacteria than the mice that survived 8 weeks, even though the infection in the surviving mice had progressed approximately 3 weeks longer.

We next compared pulmonary bacterial load with the terminal body weight at the time of euthanasia for individual mice (Fig. 4C). Mice were euthanized at 25 (*n*=10), 35 (*n*=15) and 45 (*n*=14) days post-infection. Early-mortality mice (*n*=17) were euthanized between 30-41 days post-infection based upon IACUC morbidity guidelines. Pulmonary bacterial load inversely correlated with mouse body weight, which proved to be a reliable indicator of disease severity in conjunction with physical parameters (e.g. dyspnea, ruffled fur) and behavioral characteristics (e.g. hunched appearance, lethargy). Initial deviations in individual animal body weights could be observed by 20 days following LDA, and were predictive of disease severity and early mortality (data not shown).

### Lesion burden stabilized by 45 days following infection

**Fig. 5.**
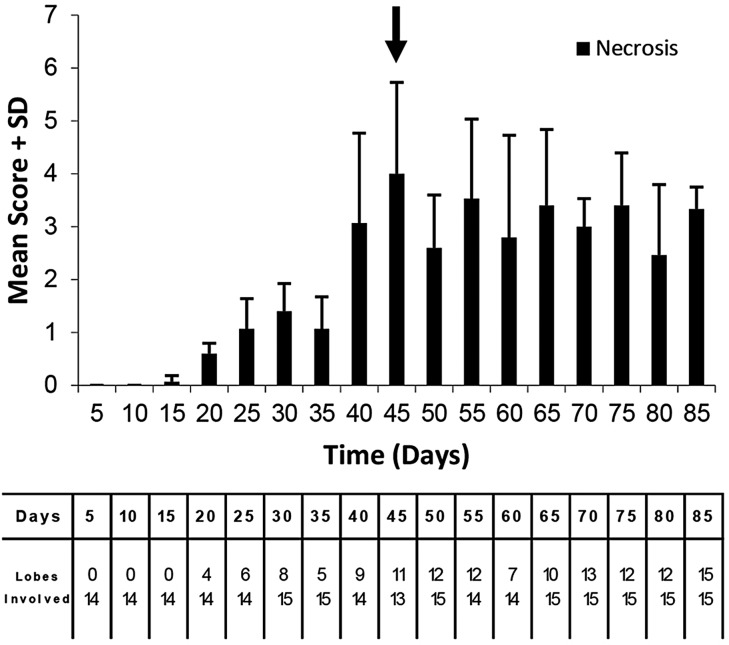
**Lung lesion burden rapidly increased until 45 days in *M. tuberculosis-*infected C3HeB/FeJ mice.** The data represent the mean lung lesion burden on a scale from 0 to 5, plus the standard deviation from three animals at each pre-determined end-point. Arrow indicates the transition where lesion scores stabilize. The data underneath the graph shows the number of lung lobes that had detectable lesions (top value) compared to the total number of lung lobes evaluated. All samples were histopathologically evaluated by a board-certified veterinary pathologist in a blinded manner.

The progression of pulmonary disease in C3HeB/FeJ mice following LDA infection was characterized by collecting mice for histological examination at 5-day intervals through 85 days of infection (Fig. 5).

The first evidence of inflammation related to *M. tuberculosis* infection occurred at 15 days following infection and pulmonary lesion burden increased with each subsequent time point through 45 days of infection, with a particularly abrupt transition between 35 and 40 days of infection. After 45 days, the pathology in the lungs of C3HeB/FeJ mice as measured by total lesion burden scoring began to stabilize. The 35- to 40-day time period coincided with the 28- to 45-day window of mortality observed in the survival experiments (Fig. 1B). Beyond 45 days of infection, there was no significant increase in the number of lung lesions and it seemed that the pathology in the lungs of C3HeB/FeJ mice began to stabilize, although individual lesions continued to increase in size and severity over time.

### Intra-mouse lesion heterogeneity was observed throughout the time course of infection

**Fig. 6.**
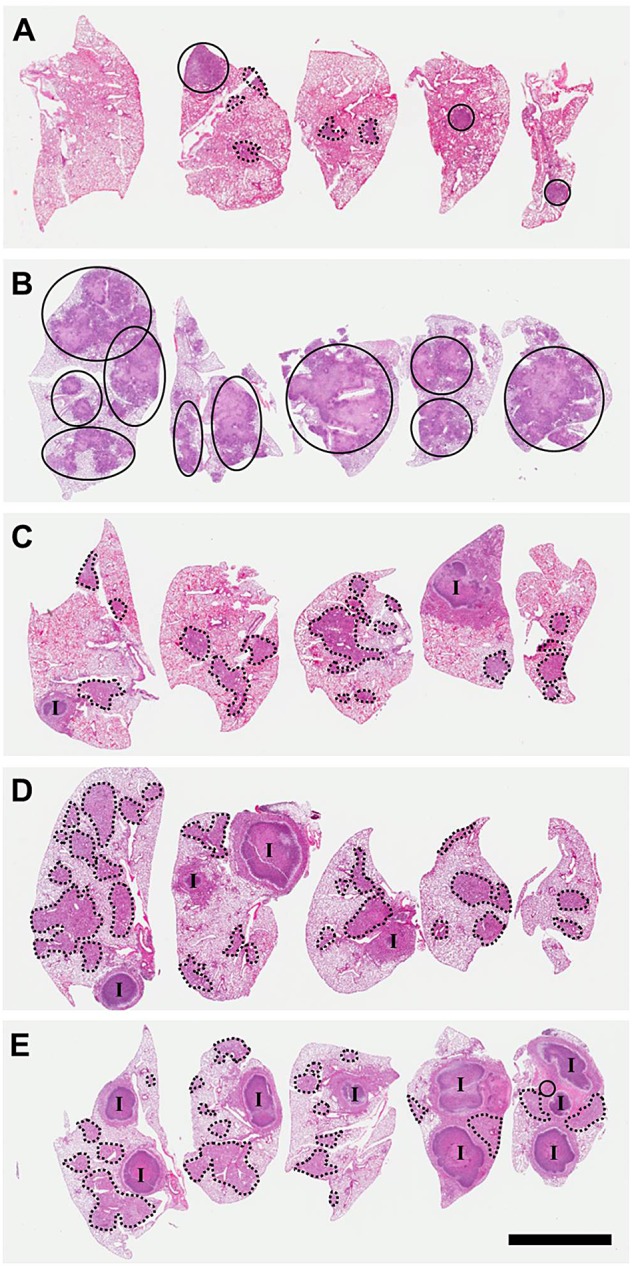
**Intra-mouse lesion heterogeneity following LDA infection.** Micrographs show all lung lobes (H&E-stained) from a representative C3HeB/FeJ mouse at 4 weeks (A), 6 weeks (C), 8 weeks (D) and 10 weeks (E) post-infection, and a representative early-mortality mouse at day 34 post-infection (B). Type I lesions (I), Type II lesions (circled regions) and Type III lesions (dashed circles) are indicated. Scale bar: 5 mm.

Owing to the presence of three lesion types, a pathological assessment was performed to determine the spatial distribution of each lesion type within each lung lobe of individual animals following LDA infection. Type I lesions were non-uniformly distributed between the five lung lobes of individual animals (Fig. 6A,C-E). Type III lesions were more numerous, generally smaller in size, and more evenly distributed between lung lobes. In the mice that predominantly had Type II lesions, nearly all of the lung lobes had evidence of such lesions (Fig. 6B).

### *M. tuberculosis* number and location varied by lesion type as visualized by SYBR Gold staining

Using the SYBR Gold acid-fast stain, we next examined the distribution of *M. tuberculosis* bacilli to determine the spatial location of the bacilli within pulmonary lesions, whether they were intracellular or extracellular, and which cell types bacilli were associated with.

**Fig. 7.**
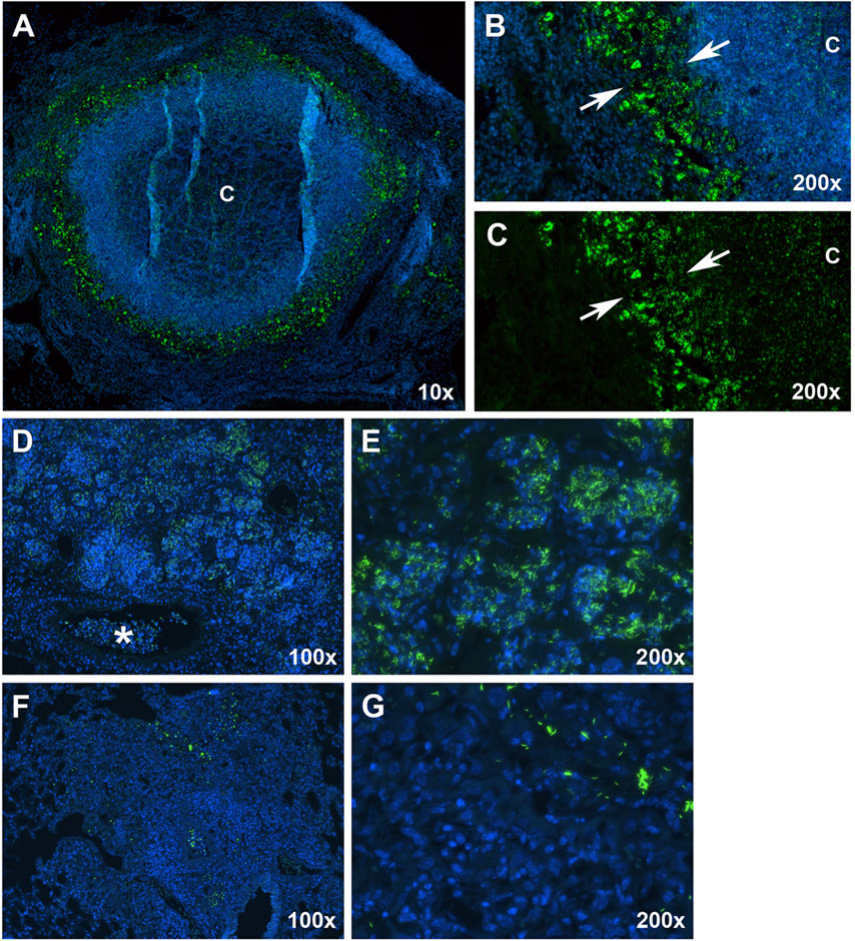
**Fluorescent acid-fast staining revealed differences in bacterial numbers and spatial distribution between the three lesion types.** (A) A mature Type I lesion with caseous necrosis, showing the distribution of bacteria using the SYBR Gold methodology. ‘C’, caseum. (B) Large numbers of intracellular bacilli were present as aggregates within foamy macrophages (arrows delineate margins). Intracellular bacilli were also present within intact neutrophils and large numbers of extracellular bacilli were found within the caseous necrotic, hypoxic caseum. (C) The identical image of panel B with the DAPI channel turned off to more easily visualize extracellular bacilli within the caseum. (D) A Type II lesion showing large numbers of bacteria present within the peripheral margins of the lesion and in the cellular debris within terminal bronchioles (asterisk). (E) Large numbers of bacilli were located within neutrophils that had consolidated alveoli. (F) A Type III lesion showing characteristically small numbers of primarily intracellular bacilli within a relatively large lesion. (G) Higher-magnification image showing that, within Type III lesions, bacilli occurred singly, or in relatively small aggregates, in the vicinity of much larger numbers of uninfected cells. Green, SYBR Gold-stained bacilli; blue, DAPI.

Examination of Type I lesions revealed a large number of intracellular bacteria, primarily confined to the rim of foamy macrophages (Fig. 7A,B, arrows). Small numbers of intracellular bacteria were confined to individual or small clusters of infected macrophages located in the periphery of the granuloma, exterior to the fibrotic capsule. In addition, abundant numbers of extracellular bacteria were localized to the central, acellular region of the caseum (Fig. 7C). Interestingly, the extracellular bacilli within the caseum stained less intensely on a per cell basis than the intracellular bacteria located within the foamy macrophage rim.

Type II lesions contained overwhelmingly larger numbers of bacilli than either Type I or Type III lesions, indicative of uncontrolled bacterial replication (Fig. 7D). Near the peripheral margin of these lesions, large numbers of bacilli were found within intact neutrophils (Fig. 7E). Within the amorphous caseous necrotic material near the center of the lesion, large numbers of extracellular bacteria were present. These bacilli stained less intensely than the intracellular bacteria located near the margins of Type II lesions. Intracellular and extracellular bacilli were also evident within the necrotic debris located within occluded terminal bronchioles (Fig. 7D, asterisk).

Type III lesions had significantly fewer numbers of bacilli per lesion, located almost exclusively intracellularly and predominantly within epithelioid-like and foamy macrophages (Fig. 7F,G). Bacteria were often present as single bacilli, or as clusters of bacilli that were typically smaller than the bacterial clusters observed in both Type I and Type II lesions.

## DISCUSSION

In these experiments, we characterized the histopathological response and the location of bacilli over time in the lungs of C3HeB/FeJ mice following aerosol infection. We identified three morphologically distinct lesion types present within the lungs of C3HeB/FeJ mice that differed with respect to cellular composition, control of bacterial replication, intracellular versus extracellular location of bacilli, degree of immunopathology, and kinetics of lesion progression. Although the progression of each lesion type was compiled by a trained veterinary pathologist, it is important to keep in mind that all histological images represent terminal endpoints and therefore lesion-type progression was inferred by examination of multiple lesions from each of the indicated time points.

Type I lesions were composed of a neutrophil-dominated central core region that degenerated over time into an amorphous, acellular caseum surrounded by a band of intact neutrophils and a distinct rim of foamy macrophages at the peripheral margin. The foamy macrophages each contained numerous intracellular bacilli, whereas large numbers of extracellular bacilli were present within the acellular caseum. This core region was encapsulated by a collagen rim deposited by fibroblasts intermixed with large numbers of epithelioid macrophages, activated macrophages, and lesser numbers of scattered lymphocytes.

Although the Type I lesions identified in C3HeB/FeJ mice more closely resembled human granulomas when compared to BALB/c mice, it should be noted that the cellular composition in the Type I lesions differed from that traditionally observed in human lesions (Canetti, 1955; Dartois, 2014). Specifically, the necrotic caseum in Type I lesions seemed to be derived primarily from neutrophils, and to a lesser degree foamy macrophages. In humans, the central core of the granuloma is believed to be composed primarily of macrophages, foamy macrophages and smaller numbers of lymphocytes, with neutrophils playing a somewhat minor role. However, in order to effectively model drug penetration into necrotic caseum and drug efficacy against bacterial phenotypes residing within hypoxic microenvironments, the source of the caseous necrotic debris might not be the most important factor. The presence of hypoxic, caseous necrotic material itself and the disintegration of vasculature surrounding the granuloma might be sufficient to mimic the conditions within human granulomas to afford realistic drug activity assessments. Studies are currently in progress to quantify drug concentrations within caseous necrotic granulomas from C3HeB/FeJ mice using HPLC LC-MS/MS and MALDI-MSI (Prideaux et al., 2011) to address this question.

The Type II lesions in infected C3HeB/FeJ mice presented as a fulminant granulocytic pneumonia. These lesions resembled Type I lesions in that they were predominantly composed of neutrophils; however, Type II lesions lacked the fibrotic encapsulation and the cellular organization found in Type I lesions. Importantly, Type II lesions contained very few if any detectable lymphocytes. These lesions were rapidly progressive, led to consolidation of large areas of lung parenchyma, and negatively impacted survival. Mice began displaying signs of morbidity (substantial enough to warrant euthanasia) between 28-45 days following aerosol infection. All of the mice that succumbed during this time had predominantly Type II lesions and extensive pulmonary consolidation occupying the majority of viable lung parenchyma. Thus, the characteristic window at 28-45 days after infection seemed to represent the amount of time necessary to generate and recruit enough neutrophils into the lung to compromise respiratory function. The Type II lesions observed in C3HeB/FeJ mice closely resembled polymorphonuclear alveolitis occasionally observed in humans with TB, as described by Canetti (1955).

Type III lesions in C3HeB/FeJ mice were very similar to lesions found in BALB/c mice following aerosol infection. These lesions were composed predominantly of epithelioid and foamy macrophages with large numbers of lymphocytes present in cellular aggregates and interspersed throughout the lesion. Bacteria within these lesions were few in number and primarily located intracellularly within epithelioid and, at later stages, foamy macrophages. Individual cellular necrosis was evident primarily at the later stages of infection, and was predominantly the result of the disintegration of foamy macrophages. Caseous necrosis was never observed associated with this lesion type.

Cavitary lesions play a crucial role in human TB because the bacilli within these lesions are extremely resistant to drug therapy and the host immunological response, and their presence correlates with a poor clinical prognosis (Canetti, 1955; Dartois and Barry, 2013). The interior of the cavity is also the site for rapid bacterial proliferation coupled with ready access to the airways, which facilitates TB transmission. Although cavitary lesions occur naturally in C3HeB/FeJ mice following aerosol infection (Driver et al., 2012), it is generally a rare event. A current focus in our laboratory is to develop strategies to make cavitary lesions more reproducible in C3HeB/FeJ mice.

An important observation of comparing all three lesion types was that the number of lymphocytes was inversely proportional to the bacterial burden. As such, these three lesion types represented differing levels of host immunological control within the lung following infection. Type III lesions contained abundant lymphocytes and controlled bacterial replication more efficiently, maintained bacilli within intact foamy macrophages for the longest period of time, and progressed at a rate slower than the other two lesion types. These observations are consistent with initiation of a strong adaptive immune response capable of controlling bacterial replication and limiting host immunopathology. In contrast, Type II lesions contained few if any identifiable lymphocytes, failed to control bacterial replication, and rapidly progressed to a state in which consolidation of viable lung parenchyma negatively impacted animal survival. This lesion type reflected a failure of the host to initiate a robust protective immune response within the lung. Severe pulmonary neutrophilia might represent an ineffective and immunopathological compensatory action in the absence of strong adaptive immunity, facilitating uncontrolled bacterial replication and ultimately favoring the bacteria. Type I granulomas represented an intermediate level of host adaptive immunity, where rapidly progressive neutrophilic lung consolidation was held in check by the host pro-fibrotic response; however, the host response was unable to reverse the pathological progression once the granuloma became established. Lymphocytes were clearly visible within the peripheral margins of Type I lesions; however, the number of such cells was much less than that seen in Type III lesions. Although the number of bacteria continued to increase over time, the rate of increase was slower than that seen in Type II lesions. It is important to understand that, although each of the described lesion types differed in pathological severity, level of host control of bacterial replication, and damaging neutrophilic infiltration, all three lesion types continued to increase in size and degree of inflammation over time. The rate of increase was most rapid for Type II lesions and slowest for Type III lesions. Once established, these lesion types did not seem to interconvert over time. C3HeB/FeJ mice that were infected with a less virulent strain of *M. tuberculosis* that initially produced only Type III lesions did not exhibit caseous necrosis, even 20 weeks following aerosol infection (data not shown).

The role that neutrophils play during TB infection is controversial. Previous reports have identified an increased susceptibility (Pedrosa et al., 2000; Seiler et al., 2000) and a delayed initiation of adaptive immunity following depletion of neutrophils (Blomgran and Ernst, 2011), although other studies have ascribed a damaging role in which neutrophils mediate substantial immunopathology (Major et al., 2013; Marzo et al., 2014; Subbian et al., 2013). Neutrophils might indeed play an important early role transporting antigen to the draining lymph nodes and providing warning signals to dendritic cells through the uptake of apoptotic debris containing viable *M. tuberculosis* bacilli (Alemán et al., 2007; Davis and Ramakrishnan, 2009). However, uncontrolled neutrophil accumulation within the lung after this early beneficial window markedly increases pulmonary pathology by consolidation of alveolar space, release of damaging neutrophilic contents [reactive oxygen species (ROS), proteases, etc.] and release of products that contribute to sterile tissue inflammation processes. Our results clearly support an immunopathological role for neutrophils by mediating tissue destruction, which resulted in significant early mortality. Importantly, the massive neutrophilic response represented a highly permissive environment supporting bacterial replication, both within the neutrophil and within the acellular necrotic debris within the airways. This result is consistent with a study that demonstrated that *M. tuberculosis* containing an intact RD1 locus was able to subvert neutrophil-mediated killing by inducing neutrophil necrosis through a ROS-dependent mechanism (Corleis et al., 2012).

It is becoming increasingly apparent that neutrophils might play a prominent role during reactivation (specifically recrudescence) of human TB. Neutrophils represent the predominant cell type found in bronchoalveolar lavage and in sputum of individuals with active TB, and often contain large numbers of actively replicating bacilli (Eum et al., 2010). The identification that interferon-inducible transcripts from human neutrophils correlated with disease severity suggests that neutrophils could represent a biomarker for active TB or latently infected individuals likely to progress to active disease (Berry et al., 2010). Animal models that display a robust neutrophilic response provide insight into the pathological role of uncontrolled neutrophilia, and might facilitate testing of immunotherapeutic strategies to suppress neutrophil influx to limit pulmonary immunopathology during reactivation of TB disease.

Another inbred mouse strain (CBA/J mice) that exhibits caseous necrotic pulmonary lesions following aerosol infection also displays a correlation between rapid weight loss, clinically observable signs of morbidity, and extensive neutrophilic infiltration of lung parenchyma (Major et al., 2013). Interestingly, the authors also observed a bimodal survival distribution in response to aerosol infection. Although the immunological basis for the increased susceptibility of CBA/J mice is not known, the observation that two dissimilar, genetically inbred strains of mice (C3HeB/FeJ and CBA/J) respond to standardized aerosol infection with divergent outcomes suggests a stochastic mechanism underlying the host response to the initial infectious foci within the lungs of these mouse strains.

Although we observed substantial intra-mouse variation in the spatial distribution of lesion types between lung lobes from individual animals (Fig. 6), the pathological assessments and bacterial CFU determinations were remarkably similar between mice (Fig. 1A). The understanding that three distinct lesion types emerged in the lungs following LDA infection helps to explain these results. To minimize experimental bias due to the unequal distribution of lesions, our laboratory typically collects all of the lung lobes from individual animals for endpoint determinations such as histological evaluation and bacterial counts. Also, the mortality observed in these experiments reduced the inter-animal experimental variation by eliminating mice with predominantly Type II lesions. It should be noted that the use of less virulent strains of *M. tuberculosis* might not generate the same level of mortality, and could therefore result in larger experimental variation. However, even after elimination of the majority of Type II lesions, the variability of the surviving C3HeB/FeJ mouse population was still somewhat higher than that typically observed in BALB/c mice, which only present with Type III lesions. To maintain the statistical power of experiments, extra mice are now routinely added to experimental and control groups (Lenaerts et al., 2004). Although the increased number of animals and the analysis of all lung lobes adds to the difficulty of working with this animal model, these measures mitigate intra-animal variability due to the unequal distribution of lesions and greatly improve experimental reproducibility.

Although inter-animal variability has a negative impact upon statistical differentiation between groups of experimentally treated animals, a mouse model such as BALB/c mice that represents only one lesion type present within the spectrum of human disease will by necessity have decreased inter-mouse variability and increased experimental statistical power, but at the expense of a more realistic and complete representation of human disease. Sacrificing realism for increased reproducibility might ultimately compromise drug development efforts when the results obtained from such models are applied to human patients displaying the full spectrum of lesion types. Following the characteristic window of mortality, the C3HeB/FeJ mouse model seems to offer a reasonable compromise by representing two distinct lesion types (solid cellular, non-necrotizing lesions and caseous necrotic granulomas) important for assessing drug activity and the pharmacokinetic profile, while still maintaining experimental variation within useful limits to discern statistical differences between drugs.

Based upon results presented in this paper as well as other experiments performed in our laboratory, important caveats for optimal utilization of the C3HeB/FeJ mouse model include: (1) collection of all lung lobes to mitigate the variability in the distribution of lesion types, (2) use of larger numbers of mice per group to increase statistical power of the experiment, (3) allowing sufficient time for the caseous necrotic pathology to develop (which is dependent upon the virulence of the strain of *M. tuberculosis*), and (4) histological verification of the pulmonary pathology at the start of treatment.

Although the exact combination of factors responsible for the initiation of each lesion type remains to be elucidated, the initial number of bacteria deposited within the lung, the relative virulence of the strain of *M. tuberculosis* used, the route of infection, intrapulmonary lesion metastasis, and the level of bacterial aggregation during the aerosolization process likely play a role in modulating the ratio of lesion types observed within individual animals. As we were able to identify precursors of each lesion type by 35 days of infection, we hypothesize that the type of lesion that ultimately develops at each inflammatory focus within the lungs of C3HeB/FeJ mice is determined at the host-pathogen interface early during the course of infection. These observations are consistent with a recent paper that demonstrated that differences in the innate immune response between *M. tuberculosis* HN878- and CDC1551-infected rabbits occurred within 3 hours of aerosol infection, and that many of these differences remained at 4 weeks post-infection (Subbian et al., 2013).

Experiments are currently in progress to identify the factors that control the formation of each lesion type by direct modulation of the lung environment by vaccination and through intrapulmonary delivery of key cytokines to increase the uniformity of the C3HeB/FeJ mouse model. In this way, we hope to decrease the incidence of Type II lesions and direct the model exclusively towards Type I and Type III lesions. A recent report identified tumor necrosis factor as being important in this process (Dutta et al., 2014), because neutralization resulted in uncontrolled pulmonary neutrophilia, which resembled the Type-II-dominated early-mortality mice in this study. Ultimately, a better understanding of the immunological factors that dictate the formation of each lesion type will reduce the variability inherent within this mouse model, increasing the usefulness of the C3HeB/FeJ mouse model.

One shortcoming of many animal models is that the number of bacteria visible in lung sections by acid-fast staining methods is generally small (Hoff et al., 2011; Lin et al., 2009). An important aspect of C3HeB/FeJ mice is that bacterial numbers within the lung are approximately two orders of magnitude larger than in BALB/c mice, facilitating microscopic identification of large numbers of bacteria. When coupled with the highly sensitive, photostable SYBR Gold staining methodology previously described (Ryan et al., 2014), even small numbers of bacilli can be reliably detected in lung sections. Understanding the location of bacilli within the lungs is essential for drug development. The distribution of bacteria between different lesion types and the specific location within lesions provides crucial information about the microenvironmental conditions that the bacilli experience, including oxygen tension, nutrient availability and pH, because these factors impact the metabolism and phenotype of the bacilli. Solid caseous necrotic granulomas can be especially difficult to treat because the necrotic caseum of developed granulomas can impede the penetration of some drugs, and the vascularization of these lesions is compromised owing to localized immunopathology (Dartois, 2014). Because the bacteria within the caseum are primarily extracellular, drugs that accumulate within macrophages might also be less effective.

In these experiments, we identified three morphologically distinct lesion types in the lungs of C3HeB/FeJ mice following LDA infection. Importantly, the Type I lesions in C3HeB/FeJ mice represented an environment with abundant extracellular bacilli located within the necrotic caseum, whereas Type III lesions represented intracellular bacteria within non-necrotic cellular lesions. Furthermore, we observed that these lesion types were characterized by different levels of bacterial replication, which inversely correlated with the magnitude of the lymphocyte response. We determined that animals with the most severe immunopathology presented primarily with a single lesion type characterized by a damaging, rapidly progressive granulocytic pneumonia that was ultimately responsible for the increased mortality of these mice. Using the novel SYBR Gold acid-fast staining method, we identified the spatial distribution of bacilli within each lesion type and showed that, in Type II lesions, neutrophils represented a highly permissive environment for bacterial replication.

It is important to understand how TB lesion diversity impacts bacterial physiology and pharmacokinetic properties such as drug penetration in order to accurately model and predict *in vivo* drug activity. C3HeB/FeJ mice, which present with a more realistic spectrum of lesion types compared with other strains, have the potential to allow us to identify and target subpopulations of persistent organisms, to quantify drug penetration into cellular and caseous necrotic lesion types, and to decrease the likelihood of drug resistance. Increased knowledge of these critical parameters will facilitate the rational combination of novel drugs into more effective drug regimens capable of shortening the duration of chemotherapeutic treatment.

## MATERIALS AND METHODS

### Animals

All research protocols were approved by Colorado State University's IACUC, and regulations concerning the animal use were adhered to for these experiments. Female specific pathogen-free C3HeB/FeJ mice aged 6-8 weeks were purchased from Jackson Laboratories (Bar Harbor, ME). Mice were housed in a bio-safety level III animal facility and maintained with sterile bedding, water and mouse chow. Specific pathogen-free status was verified by testing sentinel mice housed within the colony.

### Bacteria

The *M. tuberculosis* Erdman strain (TMCC 107) was used for aerosol infections of mice, and the inocula were prepared as previously described (Lenaerts et al., 2005). Briefly, the bacteria were originally grown as a pellicle to generate low-passage seed lots. Working stocks were generated by growing to mid-log phase in Proskauer-Beck medium containing 0.05% Tween 80 (Sigma Chemical Co., St Louis, MO) in three passages, enumerated by colony counting on 7H11 agar plates, divided into 1.5 ml aliquots and stored at −70°C until use.

### Aerosol infection

C3HeB/FeJ mice were exposed to an LDA infection with *M. tuberculosis* in a Glas-Col inhalation exposure system, as previously described (Kelly et al., 1996), resulting in an average of 55-62 bacteria in the lungs on the day of exposure. Five mice were sacrificed the following day to determine the number of CFU implanted in the lungs.

### Enumeration of bacterial load of lungs and spleen

At the time of sacrifice, whole lungs were aseptically removed and were used for bacterial enumeration and disrupted with a tissue homogenizer (Glas-Col Inc., Terra Haute, IN). The number of viable organisms was determined by plating serial dilutions of whole lungs homogenized in 4 ml of PBS on Middlebrook 7H11 agar plates supplemented with OADC (GIBCO BRL, Gaithersburg, MD). Colonies were counted after at least 21 days of incubation at 37°C as previously described (Lenaerts et al., 2005).

### Pathology and microscopic analysis of tissue samples

Whole lungs were collected at necropsy and fixed in 4% paraformaldehyde in phosphate buffered saline (PBS). Tissue sections were embedded in paraffin and sectioned to 5 μm thickness. Subsequent tissue sections were mounted on glass slides, deparaffinized and stained either with: H&E, Masson's Trichrome collagen stain or SYBR Gold fluorescent stain (Ryan et al., 2014) as described below. Sections stained with fluorescence dyes were visualized using a Nikon Intensilight mercury vapor lamp and scanned using a Nikon TE-I motorized microscope controlled by Nikon NIS Elements AR software (v. 4.00.01; Nikon, Melville, NY) with FITC, TRITC and DAPI filters. Light microscopy sections were visualized using an Olympus BX41 with Olympus DP70 camera controlled by Olympus DP software (Olympus, Melville, NY).

### Staining procedures

At each sacrifice, whole lung lobes were infused with 4% paraformaldehyde (EMS, Hatfield, PA), preserved for 48 h in paraformaldehyde, then washed and kept in 70% ethanol until being processed for histopathological assessment. Paraffin sections (5 μm) were stained with SYBR Gold fluorescent dye (Life Technologies, Grand Island, NY) as previously described (Ryan et al., 2014). Briefly, the commercially available dye was diluted 1:1000 in a stain solution of 0.85 M phenol in a 60% glycerol/14% isopropanol solution in distilled water. The slides were heated on a block at 65°C for 5 min and then cooled at room temperature for an additional 5 min. The tissue sections were washed with acid alcohol (0.5% HCl in 70% isopropanol) for 3 minutes, then washed with water and counterstained with hematoxyin QS (Vector Laboratories, Inc., Burlingame, CA), for 5-10 s. The excess hematoxyin was washed away with ddH_2_O and slides were subsequently stained with 4′,6-diamidino-2-phenylindole (DAPI; Sigma Chemical Co.) at 200 ng/ml final concentration for 10 min and washed again with water. Slides were mounted with Prolong Gold antifade mounting media (Invitrogen; Grand Island, NY).

Collagen was stained in the mouse tissue sections using the Masson's Trichrome Kit (American MasterTech, CA) using the manufacturer's procedure. Briefly, tissue sections were dewaxed in xylene and rehydrated through a graded alcohol series. The sections were briefly rinsed in water and heated at 56°C for 1 h with Bouin's Fluid. Slides were washed then stained with Weigert's hematoxylin. Slides were rinsed and stained with Biebrich Scarlet-Acid Fuchsin for 15 min, after which they were rinsed. Slides were stained with Phosphomolybdic/Phosphotungstic acid for 5 min and placed directly into Aniline Blue stain for 10 min, then rinsed. Slides were placed in 1% acetic acid for 3 min and dehydrated by a graded alcohol series including xylene. Slides were hard-mounted using Surgipath MM24 Mounting Media (Leica Biosystems, Richmond, VA).

### Pathological scoring analysis

H&E-stained histological sections were prepared from whole lungs of C3HeB/FeJ mice at 5-day intervals (*n*=3 mice/time point). Slides were examined by an American College of Veterinary Pathologists board-certified veterinary pathologist blinded to the treatment groups. The relative lesion burden for each lung lobe from individual mice was determined by scoring (0-5 points) using the following scale: 0=no lesions, 1=focal lesion, 2=multiple focal lesions, 3=one or more focal severe lesions, 4=multiple focal lesions that are extensive and coalesce, and 5=extensive lesions that occupy the majority of the lung lobe.

Total lung and lesion areas for all five lung lobes from individual animals were quantified using NIS Elements AR software (v. 4.00.01) to analyze scanned images of representative H&E-stained slides obtained using a ScanScope XT slide scanner (Aperio, Vista, CA) at 400×. The data were expressed as the ratio of the lesion area to the lung area for individual animals.

### Statistical analysis

The viable CFU counts were converted to logarithms, which were then evaluated using Student's *t*-test. Differences were considered signiﬁcant at the 95% level of conﬁdence.
